# Broadband radiometric measurements from GPS satellites reveal summertime Arctic Ocean Albedo decreases more rapidly than sea ice recedes

**DOI:** 10.1038/s41598-023-39877-x

**Published:** 2023-08-23

**Authors:** Philip L. Dreike, Amy K. Kaczmarowski, Christopher D. Garrett, Gregory Christiansen, Erika L. Roesler, Mark Ivey

**Affiliations:** 1 Space Ground Systems Program Group, Sandia National Laboratories, P. O. Box 5800, Albuquerque, NM 87185-MS0968 USA; 2 System Engineering and Analysis Department, Sandia National Laboratories, P. O. Box 5800, Albuquerque, NM 87185-MS0971 USA; 3 Integration, Test and Analysis Department, Sandia National Laboratories, P. O. Box 5800, Albuquerque, NM 87185-MS0971 USA; 4 Focal Plane Array and Sensor Engineering Department, Sandia National Laboratories, P. O. Box 5800, Albuquerque, NM 87185-MS0971 USA; 5 Atmospheric Sciences Department, Sandia National Laboratories, P. O. Box 5800, Albuquerque, NM 87185-MS0750 USA; 6 Geoscience Research and Applications Group, Sandia National Laboratories, P. O. Box 5800, Albuquerque, NM 87185-MS0734 USA

**Keywords:** Climate sciences, Climate change, Attribution

## Abstract

New measurements from the Arctic ± 40 days around the summer solstice show reflected sunlight from north of 80°N decreases 20–35%. Arctic sea ice coverage decreases 7–9% over this same time period (as reported by the NSIDC) implying Arctic sea ice albedo decreases in addition to the sea ice receding. Similar Antarctic measurements provide a baseline to which Arctic measurements are compared. The Antarctic reflected sunlight south of 80°S is up to 30% larger than the Arctic reflectance and is symmetric around the solstice implying constant Antarctic reflectivity. Arctic reflected sunlight 20 days after solstice is > 100W/m^2^ less than Antarctic reflected sunlight. For perspective, this is enough heat to melt > 1 mm/hour of ice. This finding should be compared with climate models and in reanalysis data sets to further quantify sea ice albedo’s role in Arctic Amplification. The measurements were made with previously unpublished pixelated radiometers on Global Positioning System satellites from 2014 to 2019. The GPS orbits give each radiometer instantaneous and continuous views of 37% of the Earth, two daily full views of the Arctic and Antarctic. Furthermore, the GPS constellation gives full-time full-Earth coverage that may provide data that complements existing limited field of view instruments that provide a less synoptic Earth view.

## Introduction

The Arctic region’s fast changing climate is causing phenomena such as the decrease of the minimum (September) sea ice extent by almost 40% since the 1970s^1–3^. Several theories have been developed to explain the more rapid rate of warming of the Arctic region (called Arctic or Polar Amplification) compared to the rest of the Earth. Likely reasons behind Arctic Amplification include^4^ “reduced summer albedo due to sea ice and snow cover loss, the increase of total water vapour content in the Arctic atmosphere, changes in total cloudiness in summer, additional heat generated by newly formed sea ice across more extensive open water areas in the autumn, northward transport of heat and moisture and the lower rate of heat loss to space from the Arctic relative to the subtropics”^5,6^. Climate model-based analysis drives interpretation and hypotheses of causes behind Arctic Amplification. Several analyses have found sea ice albedo feedbacks are likely driving Arctic Amplification^7–12^. The albedo feedback is due to (1) sea ice melting that leads to recession of the ice pack as well as (2) decreasing reflectivity or albedo of the remaining ice due to surface melt^13–18^ that changes the snow and ice surface reflectivity as well as forming poorly reflecting melt ponds. Most climate system analyses recognize sea ice recession as an important effect in reducing albedo^9,14,19,20^ but the role of reduced albedo^21–23^ of the remaining sea ice is less frequently mentioned. Recent analyses of comprehensive Arctic albedo measurements over enough years to be climatologically significant (i.e., multidecadal) appear to be few in number.

Earth-reflected sunlight measurements have been made by pixelated broadband silicon radiometers (0.4–1.0 μm, visible-to-near-infrared or VNIR) operated by the U.S. Government on seven Global Positioning System (GPS) satellites^24^ at an altitude of 20,200 km. The GPS pixelated radiometers have collected measurements since 2013 and are planned to continue until 2040. Data from these instruments may provide a valuable complement to NASA’s Clouds and Earth Radiant Energy System (CERES) project^25–27^ and other measurements for determining the Earth’s radiation balance^28,29^ by providing full-time, full-Earth coverage with multiple satellites viewing all points on the Earth. The CERES program has six radiometer packages, FM-1 to FM-6 on four satellites in nearly circular low altitude near-polar orbits. FM-1 and FM-2 are on the Terra satellite, FM-3 and FM-4 are on the Aqua satellite, both launched in 1997 in 705 km high orbits. FM-5 is on the S-NPP satellite launched in 2009 and FM-6 is on NOAA-20 launched in 2014 in 834 km orbits. At this writing (2023) Terra and Aqua may be nearing end-of-life. The CERES instruments each have three bolometer-based uniform spectral response radiometer channels: short wave (0.3–5.0 microns), window (8–12 microns) and total (0.3–100 microns). Moderate Resolution Imaging Spectrometer (MODIS)^30^ hyper-spectral imagers are on Terra and Aqua and Visible Infrared Imager-Radiometer Suite (VIIRS)^31^ hyper-spectral imagers are on Suomi NPP and NOAA-20. The MODIS and VIIRS spectral bands are similar to geosynchronous weather satellite imager bands so that CERES measurements can be transferred from CERES to MODIS/VIIRS to weather imagery to provide radiometric coverage geographically and temporally. CERES has frequent, but not continuous, polar coverage. Although the GPS radiometers have narrower spectral coverage, described in detail below, than the extensive CERES instrument suite, the GPS radiometers may complement CERES with (1) better full-Earth, full-time coverage for measuring reflected sunlight, particularly providing full-time coverage of the polar regions not visible from geosynchronous orbits and (2) multiple simultaneous views of all points on the Earth to sample angular reflectance variations.

Earth reflected sunlight was also measured with the Advanced Very High-Resolution Radiometer (AVHRR) series of instruments on U.S. National Oceanic and Atmospheric Administration (NOAA) series of polar orbiting weather satellites from 1979 to 2019^32^. Cloud products were produced from AVHRR data by the International Satellite Cloud Climatology Project (ISCCP)^33,34^.

This paper describes summertime measurements of Earth-reflected sunlight from the North and South Polar regions north and south of 80°N/S made with the GPS-based radiometers. These calibrated radiometers collect a coarse image of the full visible disk of the Earth every 12.9 min. The first application of these radiometer measurements to climate science is described here. These measurements show that the albedo of the Arctic sea ice north of 80° decreases much more rapidly, likely due to melting effects mentioned above, than the sea ice areal coverage decreases as measured using satellite-based microwave measurements^35–37^. Decrease in sea ice coverage alone is commonly identified as a contributor to Arctic amplification^38^, with limited attention to accompanying albedo decrease. Exceptions are Lindsay^39^ who reported a summertime decrease in central Arctic albedo based on AVHRR satellite measurements and Lei^16^ who studied the Beaufort Sea. Review of ISCCP and MODIS cloud coverage measurements shows that the reflectivity change is not due to cloud cover effects. The GPS measurements described here show that change in the albedo of the remaining sea ice must make a large additional contribution to Arctic amplification. The difference in Arctic and Antarctic albedo 20 days after the respective summer solstices results in Arctic Top of Atmosphere reflected sunlight more than 100 W/m^2^ smaller than Antarctic reflected sunlight, corresponding to sufficient absorbed sunlight to melt more than 1mm of sea ice per hour, if it were all absorbed by sea ice.

## Results

### Time series of polar reflectance measurements

This section presents time series measurements of sunlight reflected from the Arctic north of 80°N latitude and Antarctic south of 80°S latitude made with seven GPS Block IIF radiometers. Each radiometer pixel can be traced to an area on the Earth using the pixel number, the satellite coordinates in an Earth-Centered-Earth-Fixed (ECEF) coordinate system, and the satellite’s reported pitch, roll, and yaw angles. The satellite coordinates are determined from the satellite orbital parameter data. In this calculation, the Earth is assumed to be a perfect sphere. The pixels that project to locations north of 80°N latitude or south of 80°S latitude are selected from the datasets taken at the first southern and northern orbital extreme each day. Pixel currents are summed and converted to black body equivalent irradiance at the radiometer aperture. The most uncertain part of the process is the conversion from pixel current to black body equivalent irradiance that assumes directionally uniform (Lambertian) reflectance and uses an estimate of the spectral variations of atmospheric transmission and surface reflectance as described in the Methods section. Irradiance is back projected to Earth to determine Top of Atmosphere (ToA) reflected sunlight. The incident power on the radiometers as plotted below is an upper bound, and the uncertainty factors differ for the Arctic and Antarctic.

**Figure 1:**
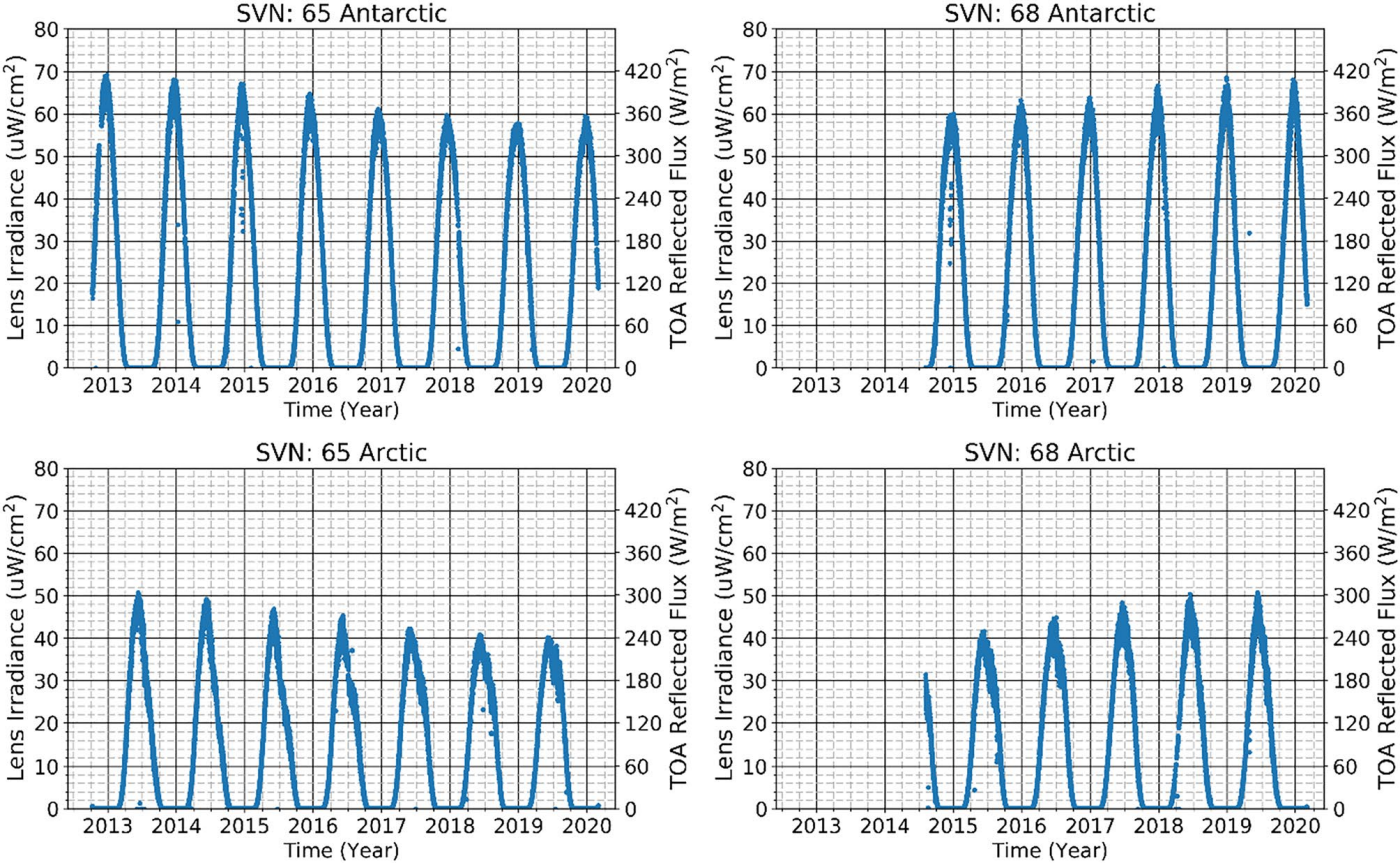
Daily Antarctic and Arctic reflected light measurements vs. time. The left-hand axes of the plots show irradiance at the radiometer apertures and the right-hand axes show that irradiance projected back to the top of the atmosphere. (Upper pair) Plot of GPS radiometer pixel measurements summed over the pixels viewing south of − 80° for each SVN with full view of the Antarctic, i.e., the satellite sub-point is south of − 53°. (Lower pair) Corresponding sum for pixels viewing north of 80°. i.e., the satellite sub-point is north of 53°.

The upper (lower) frames of Fig. 1 show the daily irradiance at the radiometer aperture from Antarctica south of − 80º latitude (Arctic north of 80°) at the times when Satellite Vehicle Numbers (SVN) 65 and 68 are near the southernmost (northernmost) point in their orbits (south/north of −/ + 53°). The peak values of radiometer irradiance are fairly consistent from year to year for the two satellites in the range of 60–70 (40–50) μW/cm^2^. As described in the Methods section, the year-to-year decrease in the SVN65 signal and the increase in the SVN68 signal appear to be due to some combination of changes in (1) the satellite longitude from year to year, which affects the view of Antarctica; and (2) the time of day of minimum latitude, which affects the solar longitude, Sun-Earth-Vehicle (SEV) angle that is the light reflection angle. The greater reflectivity of the Antarctic than the Arctic is due to the greater reflectivity of the Antarctic glacier and snow cover than of the Arctic snow-covered sea ice.

**Figure 2:**
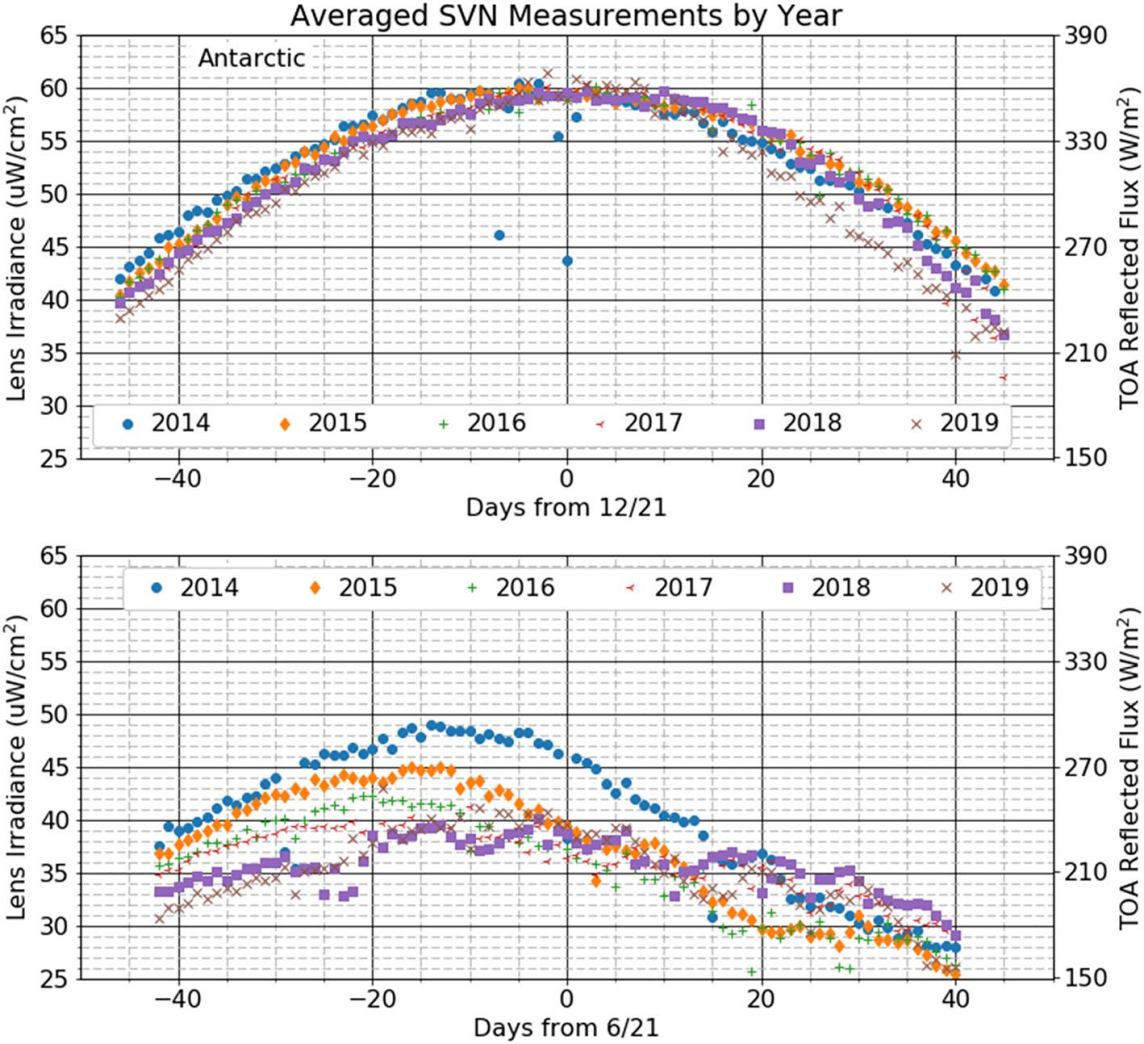
Average irradiance (left axis) and the reflected light flux at the top of the atmosphere (ToA) (right axis) values at the orbital extremes averaged over the available radiometers for each day from 40 days before the summer solstice to 40 days after in the years 2014 to 2019. Antarctic top and Arctic bottom.

Figure 2 shows the daily-averaged radiometer measurements from up to seven radiometers viewing the south and north polar regions for each year from 2014 to 2019. The generally parabolic shape of the irradiance curves is due to the changing solar illumination from the rise and fall of the Sun above the horizon around the solstice. The irradiance jitter due to satellite latitude jitter at measurement times mentioned in the Methods section is smoothed out by the averaging. The data from 2014 shows evidence of likely downlink transmission dropouts on a few days. While these dropouts can occur in the data from these instruments, the typical performance is quite stable. Year-to-year differences in the Antarctic measurement are only a few percent, suggesting good average instrumental stability as well as stable year-to-year Antarctic reflectivity. The symmetry of the Antarctic reflectivity around the solstice is striking and implies constant reflectivity of the Antarctic surface over the 80-day periods. There are three clear differences in the Arctic and Antarctic reflectivity. (1) The Arctic reflectivity is *asymmetric* around the solstice, peaking 15–20 days before the solstice; (2) the peak Arctic reflectivity varies by 30% over the years and is 15% to 35% smaller than the Antarctic reflectivity, and (3) Arctic year-to-year reflectivity differences are up to 30% for any given day, particularly near 20 days after the solstice.

Because the Earth’s orbit around the Sun is very nearly circular the solar light flux incident on the Arctic is virtually the same as on the Antarctic on a given date relative to the solstice date. Figure 2 shows the average reflected sunlight flux at the top of the atmosphere (ToA) from the Antarctic 20 days after the solstice is about 330 W/m^2^ compared with about 195 W/m^2^ from the Arctic. The difference of about 135 W/m^2^ is largely absorbed by the Arctic surface. Some of that absorbed power may be re-radiated at longer wavelengths than the radiometer is sensitive to, but the conversion process is very inefficient. The heat of fusion of ice is about 330 J/g or 3.3e5 J/mm/m^2^ (energy/thickness/area). This next rough calculation puts the heat input into perspective for sea ice melting: 135 W/m^2^ equals 4.9e5 J/m^2^/hr, sufficient to melt nearly 1.5 mm/hr of ice or about 1 m/week of medium density snow (0.16 g/cm^3^) were it all absorbed by ice and snow.

### Comparison of Arctic reflectivity with sea ice coverage

**Figure 3:**
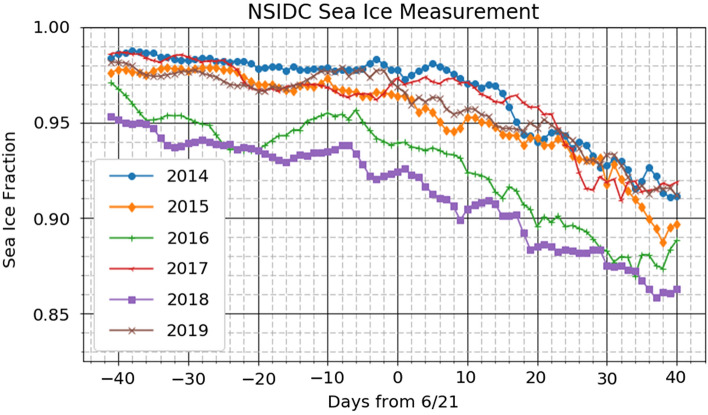
Arctic sea ice fraction by day from 40 days before the summer solstice until 40 days after the solstice from 2014 through 2019. Original data obtained from the National Snow and Ice Data Center^37^.

Passive microwave measurements of Arctic sea ice extent are tabulated and made available by the National Snow and Ice Data Center (NSIDC)^35–37^ with 25-km pixel resolution. This dataset “provides a long -term, consistently interpreted and calibrated record for studies of climate variability and change”^40^. Figure 3 shows our determination of fractional ice coverage of the Arctic north of 80ºN from 40 days before to 40 days after the summer solstice for each year from 2014 through 2019 based on the NSIDC dataset. During the winter months the Arctic sea ice completely covers the domain north of 80°N.

**Figure 4:**
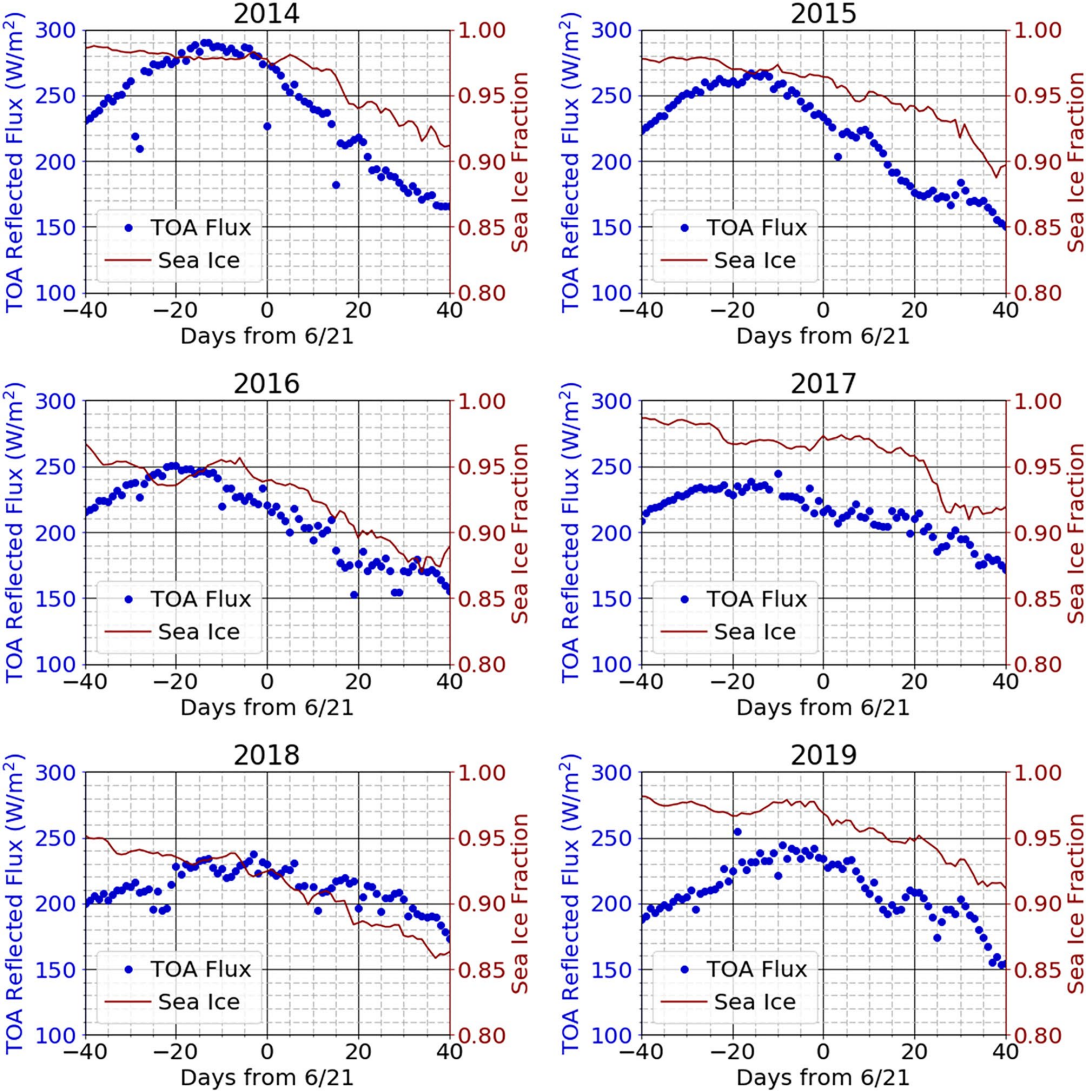
Comparison of reflected light and sea ice extent vs time from 40 days before to 40 days after the summer solstice. Arctic sea ice fraction (right axis) and average GPS-measured ToA reflected sunlight flux (left axis, from Fig. 2) day by day.

Figure 4 compares the Arctic sea ice coverage with the average Arctic GPS ToA reflected sunlight flux measurements shown in Fig. 2 day by day for each of the years from 2014 to 2019. Note that the sea ice coverage was largest in 2014 and the ToA flux was largest in 2014. The sea ice coverage was slightly smaller in 2015 than in 2014, and the ToA flux was also slightly smaller than in 2014. The ice coverage was the smallest in 2018, and the ToA flux was also generally smallest in 2018. There are also nuances in the ice coverage histories that correspond with nuances in the ToA fluxes, such as the dips in both 2014 curves at 0 and + 18 days and in the 2015 curves at about + 4 days.

The generally parabolic shape of the irradiance curves around the solstice makes it difficult to discern the change of the average Arctic albedo as the ice melts. A method is needed to compensate for the changing solar illumination of the Arctic to convert the ToA reflected flux in Fig. 4 to albedo. Our method to do this is to normalize the Arctic measurements using the Antarctic measurements as described in the methods section of this paper.

**Figure 5:**
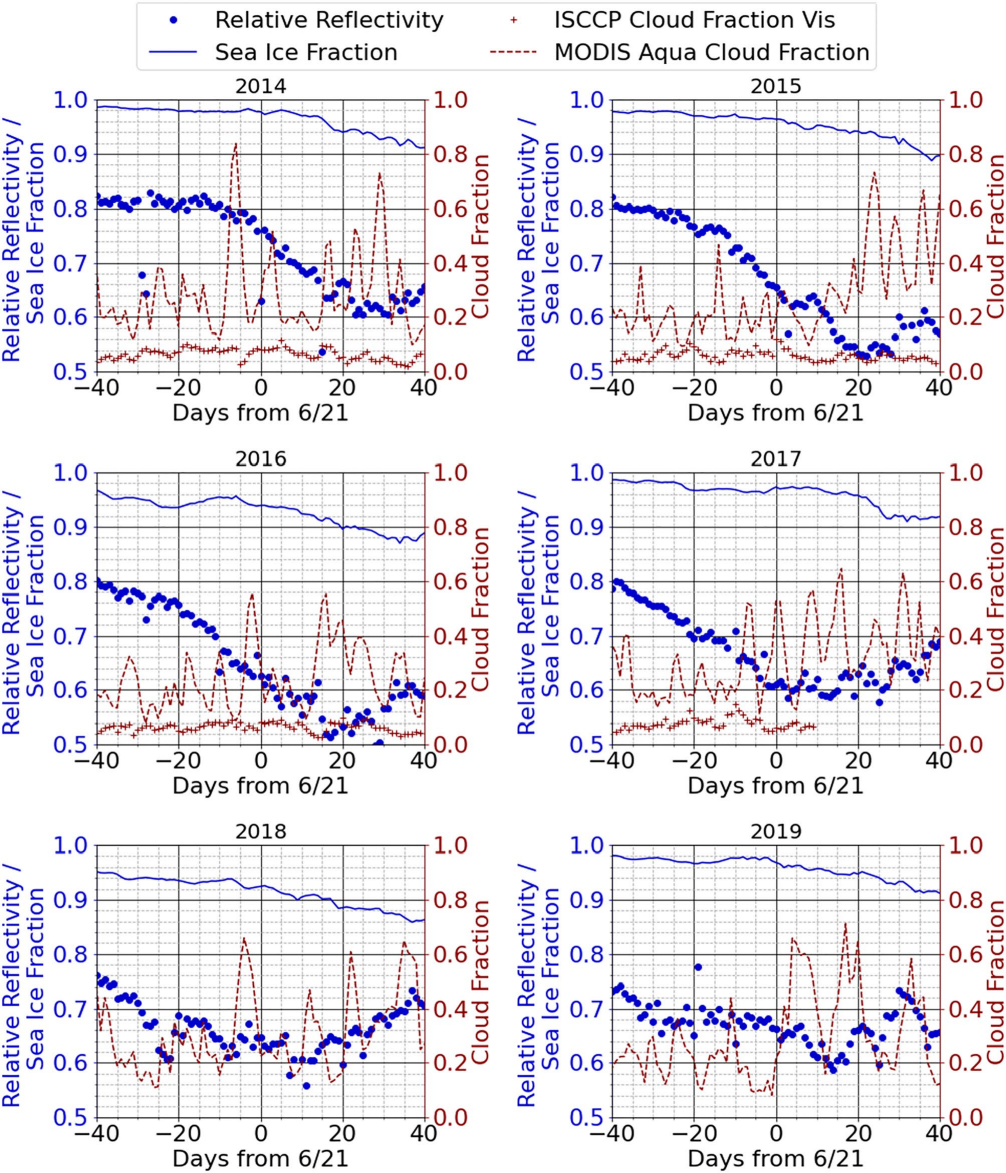
(Left axis, blue) Comparison of Arctic reflectivity relative to the average Antarctic reflectivity and sea ice fractional coverage north of 80°N for 40 days before and after the summer solstice. (Right axis, brick) Cloud fractional coverage north of 80°N from ISCCP and Modis as described in the text.

The resulting Arctic relative (to the Antarctic) reflectivity histories for 2014 to 2019 are shown in Fig. 5. The relative reflectivity history has a different shape from the shape of the sea ice history in each year. In 2014 to 2017 there are large declines in the reflectivity from days -40 to 20 and then the reflectivity stabilizes from days 20–40, all while the sea ice history shows little recession. In years 2018 and 2019 the reflectivity history rises and falls but is about the same at 40 days after the solstice as 40 days before. Before drawing conclusions, we consider possible effects of cloud cover on the measured reflectivity.

The measured reflectivity R depends not only on the reflectivity of the sea ice, but also on the reflectivity of cloud cover. Figure 5 also shows ISCCP^33,34^ “VIS” cloud fraction measurements for the years 2014 to 2017 and the MODIS Aqua^30^ cloud fraction^41^ measurements on the right hand axis. Corresponding cloud reflectivities are not available. The definition of the ISCCP VIS cloudiness^42^ is based on a reflected radiance measured with the AVHRR 650 nm channel that is in the middle of the GPS 0.4-1.0 micron spectral response. The cloudiness “clear sky” radiances (or reflectivities) across the earth are established by determining a minimum radiance for each AVHRR measurement pixel, since clouds will only increase the reflected radiance. A pixel is determined to be cloudy at a particular time if the normalized radiance exceeds the normalized clear sky radiance by a threshold that is typically 6 percentage points. The clear sky radiance for snow, sea ice or ice will be quite large so that only very reflective clouds will be recorded in the VIS cloud product. The VIS cloud coverage shown in Fig. 5 ranges from 0.05–0.15, suggesting little influence of clouds on the measured relative reflectivity, particularly when the surface reflectivity is large. There is no obvious relationship between the ISCCP VIS cloud fraction and the GPS-measured reflectivity. The MODIS cloud fraction, also shown in Fig. 5, is based on five tests^43^ of various infrared signals. The MODIS cloud fraction varies from values below 0.2 to more than 0.8 over periods of a few days. There is no obvious relationship between the infrared-based cloud cover fluctuations and GPS-measured VNIR reflectivity.

A simple model for the reflectivity R shows that the reflectivity ${\mathrm{R}}_{\mathrm{SI}}$ of the sea ice must decrease during the summer to explain the decreases observed in the 2014 to 2017 reflectivity measurements. A model for the average reflectivity R is assembled by assuming uniform or constant reflectivities R_SI_ for sea ice, R_W_ for open water and R_C_ for clouds and coverage fractions F_SI_ for sea ice, F_CSI_ for cloud-covered sea ice and F_CW_ for cloud-covered water:

$$\begin{aligned}\mathrm{R }=&\mathrm{R\, of\, clear\, view\, sea\, ice }\,= \,{\mathrm{R}}_{\mathrm{SI}}\left(1-{F}_{CSI}\right){F}_{SI}\\&+\mathrm{ \,R\, of\, cloud\, over\, sea\, ice }\,=\, {\mathrm{R}}_{\mathrm{C}}{\mathrm{F}}_{\mathrm{CSI }}{\mathrm{F}}_{\mathrm{SI}}\\&+\mathrm{\, R\, of\, sea\, ice\, under\, cloud }\,=\, {\mathrm{R}}_{\mathrm{SI}}\left(1-{R}_{C}\right){F}_{CSI}{F}_{SI}\\&+\mathrm{\, R \,of\, clear\, view\, water }\,=\, {\mathrm{R}}_{\mathrm{W}}\left(1-{\mathrm{F}}_{\mathrm{SI}}\right)\\&+\mathrm{ \,R \,of \,cloud \,covered \,water }\,=\, {\mathrm{R}}_{\mathrm{C}}{\mathrm{F}}_{\mathrm{CW}}\left(1-{\mathrm{F}}_{\mathrm{SI}}\right),\end{aligned}$$

where we ignore absorption or reflectivity by nominally clear air and higher order terms involving multiple reflections between sea ice and clouds. After assembling the five terms and some rearranging we obtain

1 $$\mathrm{R}= {(\mathrm{R}}_{\mathrm{SI}}-({\mathrm{R}}_{\mathrm{SI}}+1){\mathrm{R}}_{\mathrm{C}}{\mathrm{F}}_{\mathrm{CSI }}){\mathrm{F}}_{\mathrm{SI}}+({\mathrm{R}}_{\mathrm{W}}+{\mathrm{R}}_{\mathrm{C}}{\mathrm{F}}_{\mathrm{CW}})(1-{\mathrm{F}}_{\mathrm{SI}}).$$

Figure 5 shows that F_SI_ is near 1 so the first major term will be much larger than the second major term that we therefore neglect. The remaining first term is

2 $$\mathrm{R}\cong {(\mathrm{R}}_{\mathrm{SI}}-({\mathrm{R}}_{\mathrm{SI}}+1){\mathrm{R}}_{\mathrm{C}}{\mathrm{F}}_{\mathrm{CSI }}){\mathrm{F}}_{\mathrm{SI}}.$$

Figure 5 shows that ${\mathrm{F}}_{\mathrm{CSI }}\cong 0.1$. Since the reflectivity of clouds will be less than 1, ${\mathrm{R}}_{\mathrm{C}}{\mathrm{F}}_{\mathrm{CSI }}<0.1$ and Eq. (2) reduces to $\mathrm{R}\cong {\mathrm{R}}_{\mathrm{SI}}{\mathrm{F}}_{\mathrm{SI}}$.

Figure 5 shows that the average reflectivity of the sea ice decreases by 25–30% over the 80-day period centered on the solstice for the years 2014 to 2017. Since the sea ice fraction ${\mathrm{F}}_{\mathrm{SI}}$ only decreases by 7–9%, this means that the sea ice reflectivity ${\mathrm{R}}_{\mathrm{SI}}$ must decline by 20–25% from early May until early August. The albedo reduction is roughly three times more important than the sea ice recession in reducing total reflectivity.

This decrease in sea ice reflectivity or albedo is generally consistent with observations of melting sea ice albedo by Grenfell and Perovich^13^ near Pt. Barrow at 71°N, Perovich and Polashenski^14^ and with relatively large area observations by Lei^16^ at 74°–82° N in the Beaufort Sea. Figure 3 of Grenfell and Perovich^13^, shows the reflectivity (or albedo) of dry packed snow over cold ice was nearly 0.9 on 21 May 1979 and that it had decreased to about 0.6 on 18 June in the GPS radiometer spectral range due to surface melting and pond formation. Using Perovich’s value of 0.9 for an absolute albedo of cold snow on ice as a proxy for the Antarctic normalization, the absolute albedo in Fig. 5 decreases from about 0.67 30–40 days before the solstice (11–21 May) to 0.55–0.6 on 21 June, very similar to the decrease Perovich saw.

There are uncertainties in details of this analysis having to do with spectral transmission through the atmosphere and with spectral reflectivity that are discussed in the Methods section. Our review of the effects of cloud cover suggests that cloud cover did not play an important role in the years 2014 to 2017. (The trends in 2018 to 2019 seem different from 2014 to 2017.) However, it seems unlikely that more extensive analysis would alter the major conclusion that change in sea ice albedo is a significant contributor to increased sunlight absorption in addition to sea ice recession.

## Discussion

There are pixelated silicon photodiode-based radiometers on some of the GPS Block IIF satellites that measure Earth-reflected sunlight every 12.9 min. The radiometers view 37% of the Earth’s surface from the 20,200 km high GPS orbit. The radiometers are designed to be very stable over the 15-year GPS satellite design life. About 60% of the solar power spectrum is within the radiometer spectral bandpass (approximately 0.4 to 1.1 microns as shown in Fig. 9) so the measurement is a good proxy for the total reflected solar power.

Because of the importance of Arctic Amplification, we assembled and plotted time sequences of GPS radiometer measurements of reflected sunlight from polar regions taken ± 40 days around the Arctic and Antarctic summer solstices near the northern and southern extremes of the GPS orbits near 55°N and 55°S latitude. The pixelated measurements were limited to polar regions north of 80°N south of 80°S and south of 80°S to focus attention on the Arctic sea ice reflectivity. The central Antarctic ice sheet is stable through the summer while the Arctic sea ice melts significantly during the summer. The daily peaks of the radiometer measurements rise and fall as the peak Sun position rises and falls above the horizon around the summer solstice. The Antarctic measurements are nearly symmetric around the December 21 solstice for each of seven radiometers each year from 2014 to 2019. There are small changes from year-to-year with the largest differences between years for individual radiometers being about 12%. The changes seem more likely due to changes in Sun and satellite positions over the years and anisotropic reflectance than calibration drift or reflectance changes. The corresponding reflected light measurements vs. day from the Arctic peak 10–20 days before the June 21 solstice have much more scatter and structure than the Antarctic measurements. The peak Arctic reflected light is also ~ 20% less bright than the peak Antarctic reflected light. The day-to-day changes in Arctic illumination were normalized out using the Antarctic measurements to obtain an Arctic reflectivity history for each of the summers from 2014 to 19. The normalized reflectivity of the Arctic declined 20–35% in different years during the 80-day observation period. This defect in Arctic sunlight reflection compared with the Antarctic sunlight reflection is sufficient to melt more than 1 mm of ice per hour 20 days after the solstice, or nearly 1 m of snow per week. Review of ISCCP VIS cloud coverage measurements indicates that the reflectivity decrease is not due to changes in Arctic cloud cover.

Finally, we compared the daily Arctic reflected light with the daily microwave measured Arctic sea ice fraction from 2014 to 2019. While the ice coverage decreased by 7–9% from 40 days before the solstice until 40 days after the solstice the reflected light irradiance at the radiometers declined from 20 to 35% showing that not only does the Arctic ice coverage fraction decrease in the summer but the reflectivity of the ice also decreases on average by ~ 20%. That the sea ice albedo (reflectivity) decreases during the summer is a significant finding that should be widely appreciated and deserves incorporation into model-based studies of Arctic Amplification.

## Methods

### Using Antarctic reflected light measurements to normalize Arctic illumination

This section describes our method to convert “ToA Reflected Flux” in Fig. 4 to “Relative Reflectivity” in Fig. 5. Flipped across the equator, the geometry of summer Antarctic solar illumination and GPS view is nearly the same as the geometry of summer Arctic illumination and GPS view. Because the Antarctic ToA flux is symmetric around the solstice, we conclude that the parabolic shape is due only to the solar illumination change as the sun moves up from the horizon and back down. We use the average Antarctic reflected light history to remove the geometrical dependence of the solar illumination in the Arctic. We fitted a fourth-order polynomial to the Antarctic illumination and used it to remove the solar-angle dependence of the Arctic reflectance. The result is a plot of relative albedo vs. time. This procedure assumes that (1) the albedo of the Antarctic is constant, so that (2) the irradiance change at the satellite is due entirely to the change in solar illumination, and that (3) the Arctic and Antarctic illumination time histories are the same or at least proportional to one another.

Using pseudo equations:

3 $$\begin{gathered} {\text{Irradiance}}\,\sim \,{\text{Reflectivity}}*{\text{Illumination}} \hfill \\ {\text{Illumination}}_{{{\text{Arctic}}}} \,\sim \,{\text{Illumination}}_{{{\text{Antarctic}}}} \hfill \\ {\text{Reflectivity}}_{{{\text{Arctic}}}} \,\sim \,{\text{Irradiance}}_{{{\text{Arctic}}}} {\text{/Illumination}}_{{{\text{Antarctic}}}} \hfill \\ \end{gathered}$$

### The GPS constellation and radiometer data reduction

**Figure 6:**
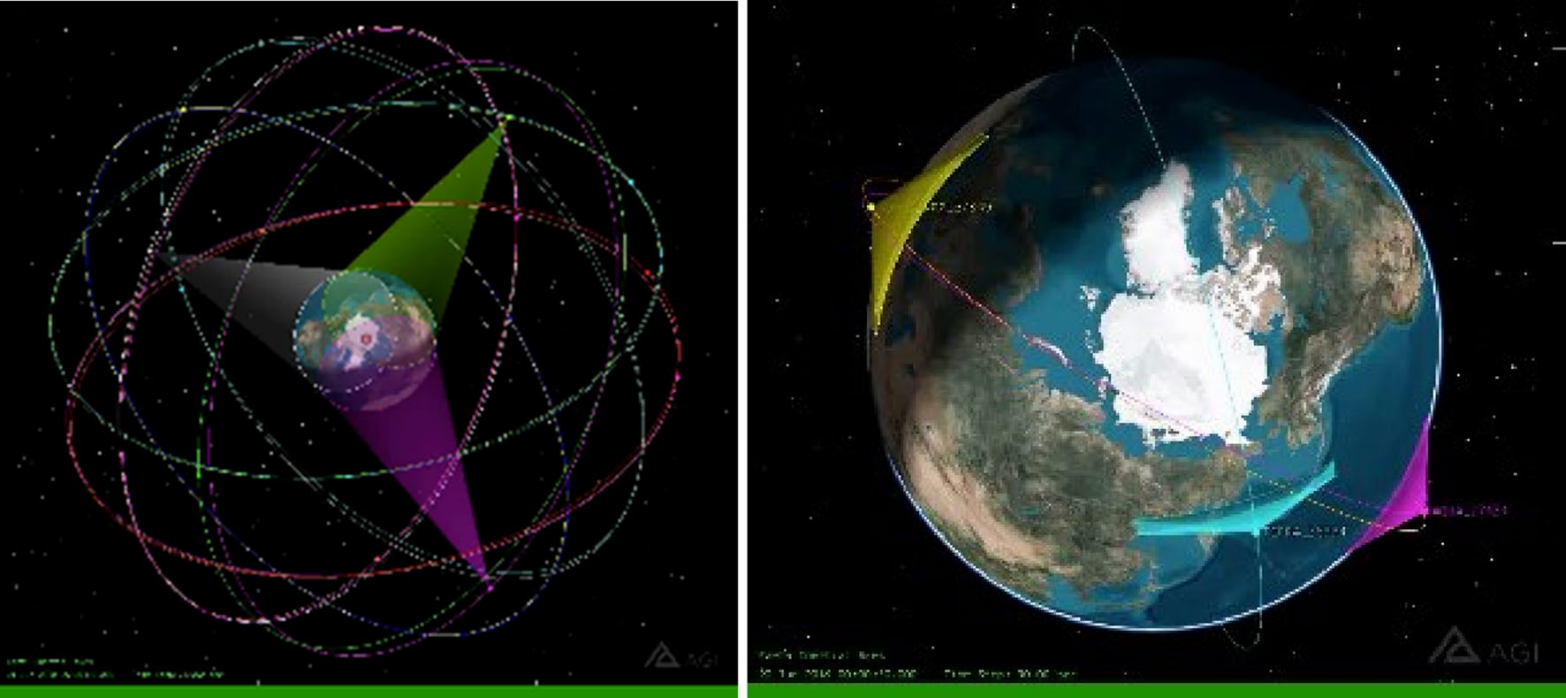
Different satellite views of the Earth. (Left) Views of the Arctic from the 20,200 km altitude GPS constellation with cones drawn showing the fields of view for three satellites with simultaneous views of the North Pole (red dot). (Right) Fan-shaped views of the Earth from 800 km altitude orbits of satellites carrying CERES payloads. The constellation of GPS satellites gives a very synoptic view of the Earth. (Figure prepared with STK^44^).

Figure 6 shows the nearly circular GPS satellite orbits^24^ at an altitude of 20,200 km in six orbital planes inclined at about 55° to the equator with an orbital period of about 11:58:02 h. There have been several blocks (versions) of satellites comprising the constellation since 1978. Beginning with Block IIA in 1990 some satellites have been equipped with silicon band radiometers. The high orbit provides each radiometer a view of 37% of the Earth and the 55° inclination gives each satellite a good view of the Arctic and Antarctic twice each day as shown in Fig. 6. This high orbit and comprehensive Earth coverage of GPS provides a more synoptic view of the Earth than is available to the CERES instruments on low Earth orbit satellites as shown on the right of Fig. 6.

**Figure 7:**
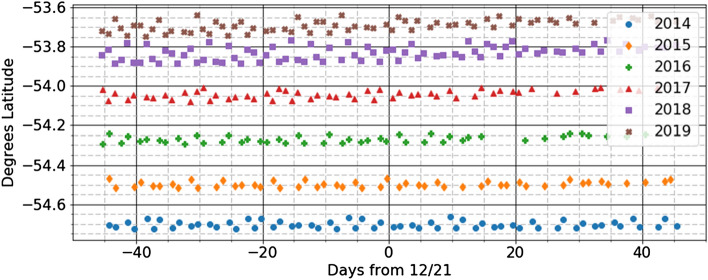
SVN65 orbit latitude at the times its Antarctic radiometer measurements were taken that are included in the averages shown in Fig. 2.

The GPS satellite orbits are optimized for the main navigation mission and each point on the Earth is typically in view of 8–10 GPS satellites at all times. Each day the time of revisit is 4 min earlier than the previous day; the solar illumination of the Earth as seen from GPS changes slowly from day to day and significantly over longer periods of time. This paper presents measurements collected near the southern and northern extremes of the orbits when the orbital subpoints are close to 55° south or north. Figure 7 shows the sub-point latitudes at which measurements were taken by SVN65 at the southernmost extreme of its orbits in the years 2014 to 2019. Because the radiometer measurements are stored in memory at approximately 12.9-min intervals that do not divide the orbital period evenly there is a small day-to-day scatter in the collection latitudes. The collection latitudes for the Antarctic measurements included in Fig. 2 from SVN65 are shown in Fig. 7. So, the satellites reach this southernmost point in their orbits at different times each day and from year to year and the longitude of the Sun is differs for all measurements.

**Figure 8:**
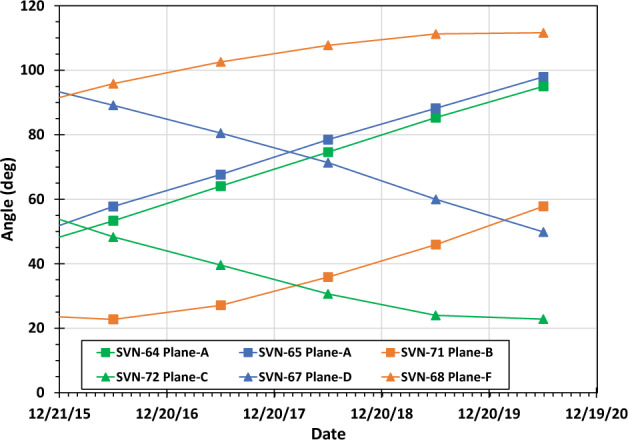
Angle from the Sun to the South Pole to the satellite when six satellites reached their minimum latitude on their first orbit of the day on December 21 in the years 2015 to 2019 and as forecast for 2020 (data from STK^44^).

Figure 8 shows the Sun-South pole-vehicle angles when six satellites reached their minimum latitude on their first orbit on December 21 in the years 2015 to 2019 and as forecast for 2020. These angles vary widely between satellites and have different variability from year-to-year for each satellite. The difference in the Sun-pole-vehicle angle has an influence on the reflected sunlight due to reflection anisotropy. These orbital effects are not taken into account in this paper and are a likely contributor to the year-to-year variations and the day-to-day scatter in the data we reviewed such as in Figs. 1 and 2. Due to the orbital period of the GPS orbits and the measurement increment time (12.9 min), data from these satellites will not exhibit the high level of measurement repeatability seen with other climate-focused satellites. This adds to the complexity of using the data, but as demonstrated in this paper does not prevent the instruments from providing insight into climate activities. Additionally, the orbital variations and views from multiple satellites at different times will provide a valuable opportunity to evaluate reflection anisotropy and its impact on the Arctic radiation balance.

**Figure 9:**
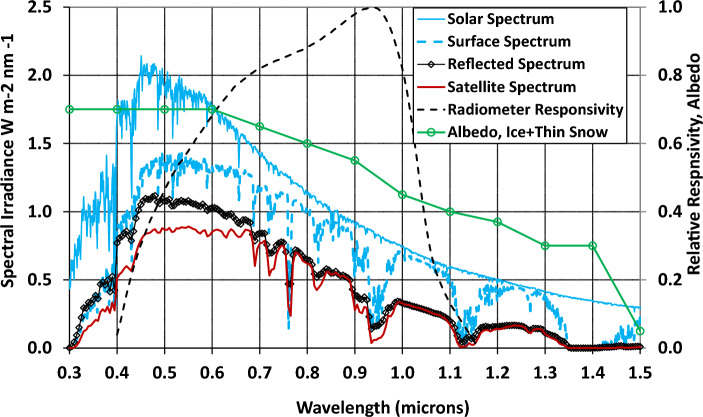
On the left axis, the solar spectrum^45^, a surface spectrum, an estimate of the spectrum reflected by pack ice with a thin cover of melting snow, the spectrum reaching the satellite and on the right axis the GPS radiometer relative responsivity and an estimate of the Arctic albedo based on Grenfell’s data^13^.

The radiometers are designed to be very radiation hard and temperature-stable so they are expected to have very stable *responsivity* over the roughly 15 year lifetime of the satellites. (Responsivity is the relationship between the light power at a given wavelength striking the lens and the current that the silicon detector produces.) We estimate the absolute calibration uncertainty of the spectral responsivity shown in Fig. 9 to be ± 10% in amplitude but that the major uncertainty is in the responsivity amplitude as opposed to the shape of the responsivity vs. wavelength. Responsivity variations across the pixel arrays are less than ± 10%.

Silicon photodiodes are essentially photon counters, so the detector current is proportional to the photon flux but not to the photon power. We convert the detector current to “Silicon-Power” (P_Si_) using the peak responsivity near 930 nm. P_Si_ is the amount of power that would be incident if all of the light were at 930 nm; P_Si_ < P for a solar-like spectrum that is bluer than 930 nm. Knowledge of the spectrum is required to accurately convert from P_Si_ to actual received power P. We use a conversion factor called the Inband Power Fraction (IPF)

$$IPF=\frac{{P}_{Si}}{P}= \frac{{\int }_{400nm}^{1100nm}S\left(\lambda \right) RR(\lambda )d\lambda }{{\int }_{0nm}^{\infty }S\left(\lambda \right)d\lambda } ;P= \frac{{P}_{Si}}{IPF},$$

where $S\left(\lambda \right)$ is the spectrum and $RR(\lambda )$ is the relative responsivity in Watts/Amp shown in Fig. 9.

The spectrum received by the detector is altered from the solar spectrum by the spectral dependences of atmospheric transmission from space to the ground, reflection, and transmission back to space. Figure 9 also shows (a) the extraterrestrial solar spectrum and (b) a modeled spectrum of light on a Sun-facing surface at 37°N with a clear sky with the Sun at the equator, both from the U.S. National Renewable Energy Laboratory^45^. The spectrum at 37°N shows the effects of molecular absorption and scattering on the spectrum. Both scattering and absorption will make somewhat larger spectral changes at the poles due to longer atmospheric path lengths, but this well-vetted readily available spectrum is a convenient approximation that gives insight into the effects on the spectrum. Spectrally dependent atmospheric transmission is the ratio of the ground spectrum to the extraterrestrial spectrum. There are no doubt variations in spectral albedo over the ~ 3.5 × 10^6^ km^2^ field of view in the Arctic, but some insight into the spectral albedo is given by Grenfell^13^ who measured albedo for several sea ice conditions. Broadly, while fresh-snow covered ice has an albedo of about 0.9 with little spectral dependence the sea ice albedo decreases as snow and ice melt and the decrease is larger in infra-red than visible. An approximation to Grenfell’s measurement of spectral albedo of “first year ice + dusting of snow” is shown in Fig. 9 where it is combined with the surface spectrum to produce a reflected spectrum. The reflected spectrum is transmitted to the satellite using transmission derived from the solar and surface spectra. IPF factors for several of the spectra are tabulated in Table 1 and they are near 0.57 ± 0.03. The Antarctic and Arctic Satellite IPF values of 0.55 and 0.60, respectively, were used for the data analyses shown in Figs. 1 and 2. IPF values uncertainties would seem to be in the 5–10% range. The increase in the IPF values from the solar spectrum’s value of 0.42 is due to Rayleigh scattering of blue light. Because the IPF values were computed using spectra from the Sun at a 37° zenith angle, these values underestimate the IPF value for light reaching the polar regions with the Sun at a 67° zenith value. For this reason, the detector irradiance values in Figs. 1 and 2 are upper limits on the actual values. Not included in this discussion is the dependence of the IPF values on the Sun angle that is introduced through the angular dependence of spectral transmission (e.g. bi-directional reflectivity, BDRF).

**Table 1:** IPF factors for the extraterrestrial solar, reflected and satellite spectra and for the surface spectrum propagated back up to space.

Spectrum	Solar	Arctic reflected	Arctic satellite	Antarctic satellite
IPF value	0.43	0.57	0.60	0.55

## Data Availability

The GPS constellation is owned and operated by the U.S. Space Force (USSF) and data collected by instruments on the GPS satellites is owned by USSF. The reduced radiometer data included in this report are derived from original data owned by USSF and are published with the permission of USSF. Data are however available from the authors upon reasonable request and with the permission of USSF.

## Code availability

Code will be made available with data upon request.
